# Transcriptome Profiling and Network Analysis Provide Insights Into the Pathogenesis of Vulvar Lichen Sclerosus

**DOI:** 10.3389/fgene.2022.905450

**Published:** 2022-06-17

**Authors:** Lingyan Wang, Qingqing Lv, Jiayi Guo, Jianwei Wang, Jing Pan

**Affiliations:** ^1^ Department of Dermatology, Beijing Jishuitan Hospital, Beijing, China; ^2^ Department of Obstetrics and Gynecology, Beijing Jishuitan Hospital, Beijing, China; ^3^ Department of Urology, Beijing Jishuitan Hospital, Beijing, China

**Keywords:** inflammatory dermatosis, vulvar lichen sclerosus, transcriptome profiling, differentially expressed genes, microRNAs, regulatory network, functional analysis, pathogenesis

## Abstract

Vulvar lichen sclerosus (VLS) is a chronic inflammatory dermatosis that affects female anogenital skin. Although VLS is considered a T cell-mediated autoimmune disease, the diagnosis criteria, molecular mechanism, and universally accepted therapies for this disease remain largely unresolved. To explore disease pathogenesis and potential biomarkers, we performed an RNA-Seq-based transcriptome analysis to profile the gene expression of VLS lesions. Differentially expressed gene (DEG) analysis revealed profound changes in expressions of coding genes, microRNAs, and long non-coding RNAs. Pathway and network analysis suggested that T cell activation-associated genes, including *CD3G*, *CD3D*, *CD8B*, *LAT*, *LCK*, *ZAP70*, *CCR5*, *CXCR3*, *CXCL9*, *CXCL10*, and *CXCL11*, were highly expressed in VLS, while *NR4A* family genes (*NR4A1*, *NR4A2*, *NR4A3*), whose coding products inhibit T cell activity, were significantly downregulated, suggesting heightened T cell response in VLS. Neutrophil chemoattractant genes *CXCL1*, *CXCL2*, *CXCL3*, *CXCL8*, and their cognate receptor *CXCR2* were downregulated, suggesting dampened neutrophil activity. We also found the downregulation of genes involved in cell cycle progression, including cyclins (*CCNB1*, *CCNB2*, *CCNL1*, *CCNE1*, and *CCNK*) and centrosome factors (*CENPA*, *CENPE*, *CENPF*, and *CENPN*), while microRNA-203a and let-7, microRNAs known to inhibit cell growth, were found to be upregulated. These data collectively indicate that cell proliferation in VLS is compromised. In sum, these findings comprehensively deciphered key regulatory genes and networks in VLS, which could further our understanding of disease mechanisms and point toward therapeutic strategies.

## Introduction

Lichen sclerosus (LS) is a chronic progressive dermatosis that most commonly affects the genitalia (Powell and Wojnarowska, 1999). The disease affects people of all ages and both sexes but is more prevalent in females than in males (Powell and Wojnarowska, 1999). It is known as vulval lichen sclerosis (VLS) when it affects vulva skin. VLS is marked by ivory-white plaques or patches that usually involve non-hair bearing areas of the vulva (Corazza et al., 2021). Typical histological features of LS consist of tissue destruction with epithelial atrophy, basal cell degeneration, dermal sclerosis, loss of rete ridges, rarefied sclerotic vessels, and band-like lymphocytic infiltrate (Powell and Wojnarowska, 1999; Pugliese et al., 2007). LS may proceed to malignant diseases such as squamous cell carcinoma (Pugliese et al., 2007). The exact prevalence of LS is unclear; however, it is estimated to range from one in 300 to one in 1,000 of all patients referred to a community-based dermatology department (Powell and Wojnarowska, 1999). For VLS, the prevalence is estimated to be approximately 1.7% based on patients presenting to a general gynecology practice in a 3-year period (Goldstein et al., 2005).

Despite being clinically defined for more than a century, much about LS remains unclear, and debate over its etiology, histological diagnostic criteria, and treatment persists. Immune dysregulation is one of the most widely recognized explanations for LS. According to reports, 82% of the overall cohort had at least one form of autoimmune illness (e.g., diabetes, vitiligo, alopecia areata, and localized scleroderma) (Tran et al., 2019). Accumulating evidence suggested that LS is an autoimmune disease with preference for a Th1 immune response (Terlou et al., 2012). Genetic factors have also been considered. The immunogenetic profile, in particular class II HLA antigens of the patients, may affect clinical manifestations (Marren et al., 1995; Azurdia et al., 1999; Tran et al., 2019). Besides, hormonal factors and infection have been proposed to contribute to LS development (Powell and Wojnarowska, 1999; Pugliese et al., 2007). To date, there is no definitive cure for LS, largely owing to the lack of understanding of the pathogenesis of the disease (Tran et al., 2019).

In the current study, we performed an RNA-seq-based gene expression profiling of skin biopsy samples from VLS patients to systematically interrogate disease pathogenesis at the molecular level. Differentially expressed genes were identified and further analyzed with functional bioinformatics tools. We further depicted molecular signatures and key regulatory networks of VLS, providing insights into VLS pathogenesis and potential biomarkers for disease management.

## Materials and Methods

### Patients and Samples

Six female patients with VLS and four healthy individuals were included. All patients recruited had classical signs of lichen sclerosus with histopathology confirmation. This study was approved by the Medical Ethics Committee of Beijing Jishuitan Hospital (202109-04).

After receipt of written informed consent signed by the patients and healthy individuals, vulval skin samples were obtained by punch biopsy so that it contained the layers of epidermis and dermis. Control samples were collected from healthy women who underwent cosmetic vulvar surgery. There is no statistically significant difference between the control and patient groups in terms of age. The biopsies obtained from each sample was put into sterile tubes and immediately frozen in liquid nitrogen until further analysis.

### RNA Isolation, Library Construction, and Sequencing

Frozen skin tissues were ground in liquid nitrogen and extracted using Trizol (Invitrogen, Carlsbad, CA) according to the manufacturer’s instructions. For library construction, total RNA that met the following criteria was used: the RNA integrity number (RIN) ≥ 7; 28S/18S rRNA ratio ≥ 1.5. The initial material for library construction was 1 μg of total RNA per sample. Using a NEBNext^®^ UltraTM RNA Library Prep Kit (NEB, Ipswich, MA), sequencing libraries with different index labels were created for each sample according to the manufacturer’s instructions. The library construction procedures were as follows. First, ribosomal RNA was removed using an Illumina Ribo-Zero™ Gold rRNA Removal Kit. Following that, RNA fragmentation was performed. The first and second cDNA strands were then generated consecutively. Purification of the library fragments was then performed, followed by terminal repair, dA-tailing, and adapter ligation. The library fragments were purified and digested using UNG enzyme. Finally, polymerase chain reaction amplification was used to finish the library construction.

The index-coded samples were clustered using a TruSeq PE Cluster Kit v4-cBot-HS (Illumina, San Diego, CA, United States) according to the manufacturer’s instructions using a cBot cluster generation system. Following cluster generation, the libraries were sequenced on an Illumina Hiseq X10 platform (Illumina, San Diego, CA, United States), and CapitalBioTech produced 125 bp paired-end reads (Beijing, China).

### Quality Control and Read Alignment

The FastQC program was used to acquire quality control metrics for raw sequencing reads (Patel and Jain, 2012). TopHat2 was used to map reads to the hg19 human reference genome, and Cufflinks was used to estimate mapped read counts (Ritchie et al., 2015). Values for fragments per kilobase of exon per million fragments mapped were used to determine transcript expression.

### DEG Analysis and Statistical Analysis

The Limma package of R language (v. 3.22.7) was used to conduct differential gene expression analysis (Trapnell et al., 2012). Paired t-tests were used to compare gene expression values between LS and healthy control samples. The Benjamini–Hochberg method was used as an FDR adjustment for multiple testing corrections. A threshold of FDR <0.05 was used to define statistical significance.

### Functional Analysis

Metascape (http://metascape.org) was employed to perform the gene enrichment and functional annotation analyses (Zhou et al., 2019). For the identification of pathways and regulatory networks, Canonical Pathway Analysis and Upstream Regulator Analysis were performed using the IPA software (Ingenuity Systems, Redwood City, CA, United States; Version: 42012434). STRING (https://string-db.org) was used to construct a protein-protein interaction network.

### Reverse Transcription–qPCR

To confirm the RNA-Seq results, RT-qPCR was performed on 12 genes. RNA was extracted using Trizol (Invitrogen, Carlsbad, CA, United States). The sequences of the qPCR primers used in this study are shown in Supplementary Table S1. Each RT-qPCR reaction was performed in duplicate using SYBR^®^ green method, and the mean threshold cycle (Ct) value for each sample was used for data analysis. The 2^−ΔΔCt^ method was used for determining the fold-change in the expression level, and *GAPDH* was used for normalization. All data were presented as the mean ± SD after normalization.

## Results

### Patient Information, RNA-Sequencing, and Analysis of Differentially Expressed Genes

**FIGURE 1:**
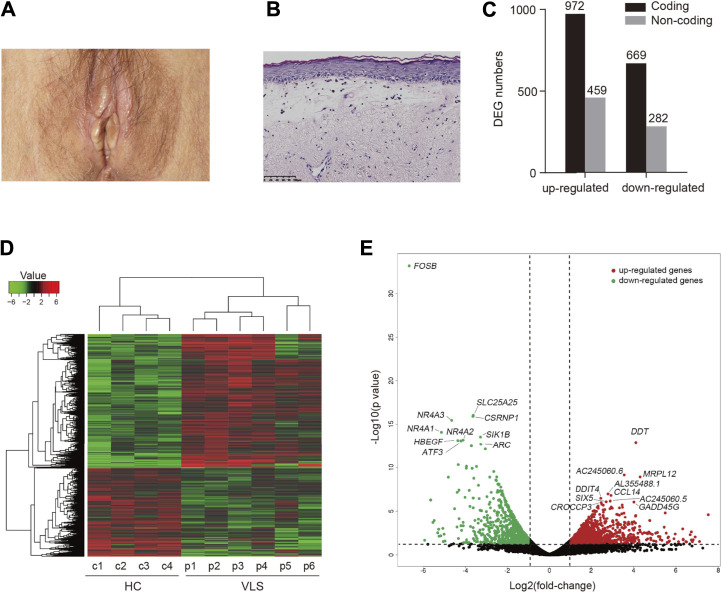
Differential gene expression analysis. **(A)** Clinical manifestation of a patient with VLS. **(B)** Histology of VLS specimen stained with hematoxylin and eosin. Scale bar, 100 μm. **(C)** Bar chart of the numbers of DEGs. **(D)** Heatmap visualization of DEGs identified in VLS *vs*. Healthy Controls (HC). **(E)** Volcano plot showing DEGs. Top ten upregulated (red) and downregulated genes (green) sorted by statistical significance (*p*-value) were labeled.

All patients fulfilled the diagnostic criteria for VLS. The demographic characteristics of the patients are detailed in Supplementary Table S2. The cutaneous manifestation of one VLS patient is shown in Figure 1A. Histological specimens of VLS stained with hematoxylin and eosin showed chronic inflammation, dermal homogenization, and loss of rete ridges, typical for VLS histopathology (Corazza et al., 2021) (Figure 1B). Detailed clinical manifestations are listed in Supplementary Table S3.

To investigate the disease mechanisms underlying VLS, we performed transcriptome profiling with biopsy samples obtained from genital lesions of the VLS patients as well as those from the genital site of healthy donors. By RNA sequencing, over 20 million reads were generated for each sample. Sequencing reads were aligned to the human genome, and differentially expressed genes (DEGs) were then identified by comparing VLS transcriptomes with those of the healthy donors. An adjusted *p*-value (FDR < 0.05) and fold change (FC) ratio (|log2FC| ≥ 1) were used to determine the DEGs. A total of 1641 differentially expressed coding genes were identified. Of those, 972 were upregulated and 669 were downregulated (Figure 1C). Moreover, a total of 741 non-coding genes were differentially expressed, comprising 459 upregulated genes and 282 down-regulated genes (Figure 1C). A heatmap of DEGs shows global transcriptome changes in individual VLS zpatients (Figure 1D). Hierarchical clustering analysis classified DEGs into two distinct groups, i.e., VLS patients and healthy donors, indicating that transcriptomes were widely and specifically regulated in patients with VLS. A volcano plot was generated to show the overall distribution of all DEGs, in which the top ten upregulated (red) and downregulated genes (green) sorted by statistical significance (*p*-value) were labeled (Figure 1E); the top 50 upregulated and downregulated DEGs are listed in Supplementary Table S4. Notably, six out of top ten downregulated DEGs, including *FOSB*, *CSRNP1*, *NR4A3*, *NR4A1*, *NR4A2*, and *ATF3*, are transcriptional regulatory factors (Figure 1E). *NR4A1*, *NR4A2*, and *NR4A3* belong NR4A nuclear receptor family involved in a variety biological process (Kurakula et al., 2014). Among top upregulated DEGs, *DDT,* and *CCL14* encode cytokines that activate mononuclear phagocytes (Tsou et al., 1998; Merk et al., 2011) (Figure 1E). Intriguingly, long non-coding RNAs (lncRNAs), including AC245060.6, AC245060.5, and AL355488.1, were most significantly upregulated (Figure 1E), indicating that lncRNAs are potentially involved in VLS pathogenesis.

### Functional Enrichment and Annotation Analysis

**FIGURE 2:**
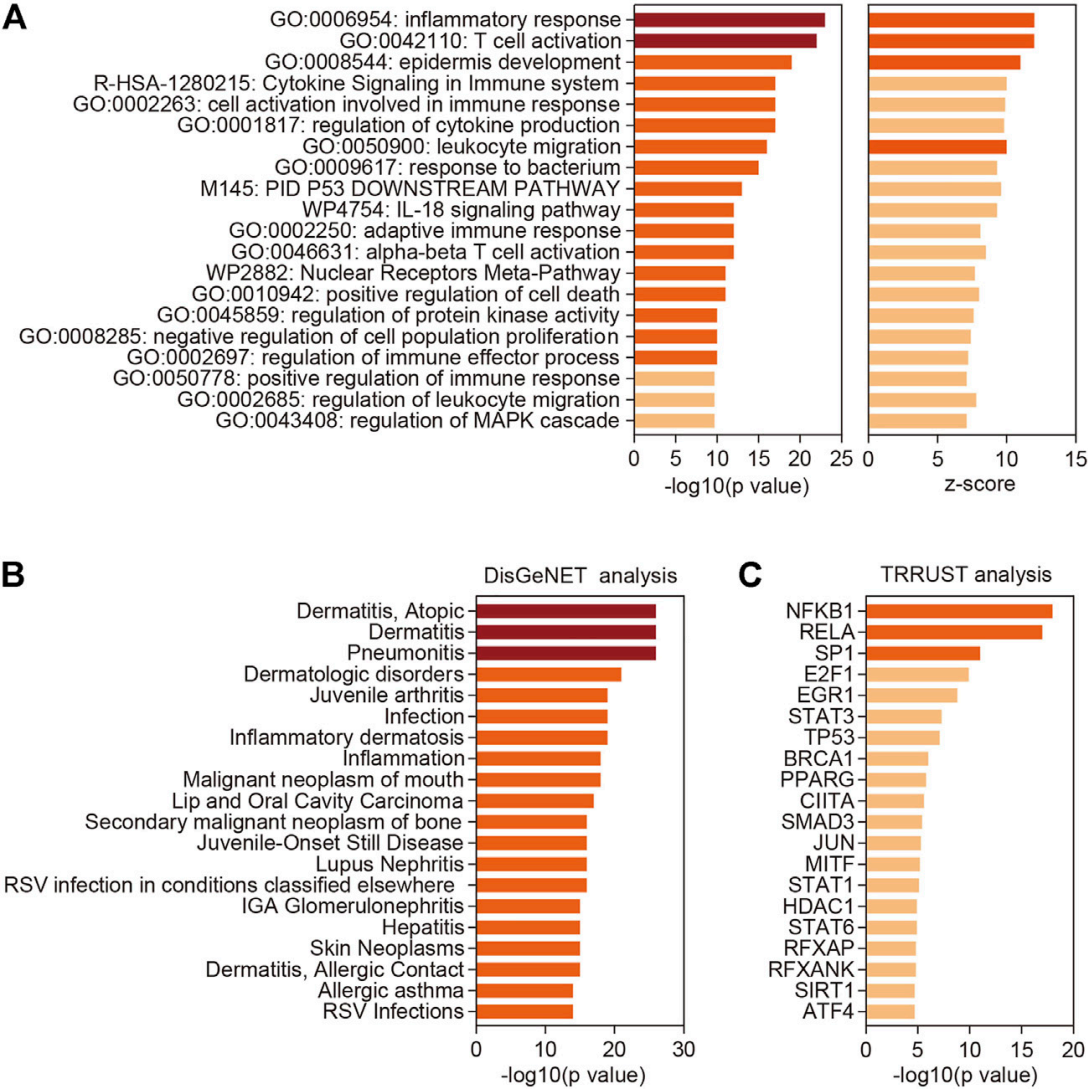
Functional enrichment and annotation analysis. **(A)** Top 20 categories enriched by Metascape analysis with all differentially expressed coding genes. *p*-values were calculated based on the accumulative hypergeometric distribution. **(B)** Summary of enrichment analysis in TRRUST (http://www.grnpedia.org/trrust/). **(C)** Summary of enrichment analysis in DisGeNET (http://www.disgenet.org/). Dark red, -log 10(*p* value) > 20; orange, 10 < -log 10(*p* value) < 20 or z-score > 10; shallow orange, -log 10(*p* value) < 10 or z-score < 10.

We then performed functional enrichment and annotation analysis of the differentially expressed coding genes (DECGs) in VLS by using Metascape (http://metascape.org) (Zhou et al., 2019), a web-based portal integrated with multiple analytical procedures. The most significantly enriched functional cluster was “inflammatory response” (Figure 2A), supporting the view that VLS is a chronic inflammatory skin disease. We also identified several other clusters related to immune responses, including “T cell activation”, “cytokine signaling in immune system”, “leukocyte migration”, “IL-18 signaling pathway”, and “adaptive immune response” (Figure 2A). Besides, categories related to cell death and differentiation, including “epidermis development”, “positive regulation of cell death”, and “negative regulation of cell population proliferation” were identified (Figure 2A).

Further, we part DECGs into upregulated genes and downregulated genes, which were then processed with Metascape. This analysis revealed that upregulated genes are mostly related to immune and inflammatory responses including T cell activation (Supplementary Figure S1A), while downregulated genes are involved in varieties of biological processes, with “epithelial cell differentiation” being the top-ranked category (Supplementary Figure S1B). T cell infiltration is a widespread phenomenon observed in vulvar and penile biopsies of LS patients (Regauer, 2005). To better understand the relevance of T cell function in VLS, we further analyzed the upregulated DECGs in the “T cell activation” category by applying a Molecular Complex Detection (MCODE) algorithm (Bader and Hogue, 2003). This analysis identified a major molecular complex composed of TCR activation-associated genes, including *CD8B*, *CD3G*, *CD3D*, *LAT*, *LCK*, and *ZAP70* (Smith-Garvin et al., 2009) (Supplementary Figure S1C), implying heightened T cell response, especially CD8^+^ T cell responses, in VLS lesions.

Next, we employed DisGeNET, a disease genomics platform (Pinero et al., 2017), to predict diseases associated with DECGs identified in VLS. Other than inflammatory dermatologic disease such as “Dermatitis, Atopic”, “Inflammatory dermatosis”, and “Dermatitis, Allergic Contact”, DECGs in VLS are linked to neoplastic disease such as “Malignant neoplasm of mouth”, “Lip and Oral Cavity Carcinoma”, “Secondary malignant neoplasm of bone”, and “Skin Neoplasms” (Figure 2B), suggesting that VLS pathogenesis share common traits with tumorigenesis. Indeed, accumulating evidence suggested that VLS can progress to malignancy (Pugliese et al., 2007). Finally, by mining TRRUST database (Han et al., 2018), we investigated transcription factors (TFs) that potentially regulate gene expression in VLS. This analysis revealed that two NFκB subunits, NFKB1 and RELA, ranked top two among all identified TFs (Figure 2C), implying the central role of NFκB in regulating gene expression in VLS.

### Canonical Pathway and Upstream Regulator Analysis

**FIGURE 3:**
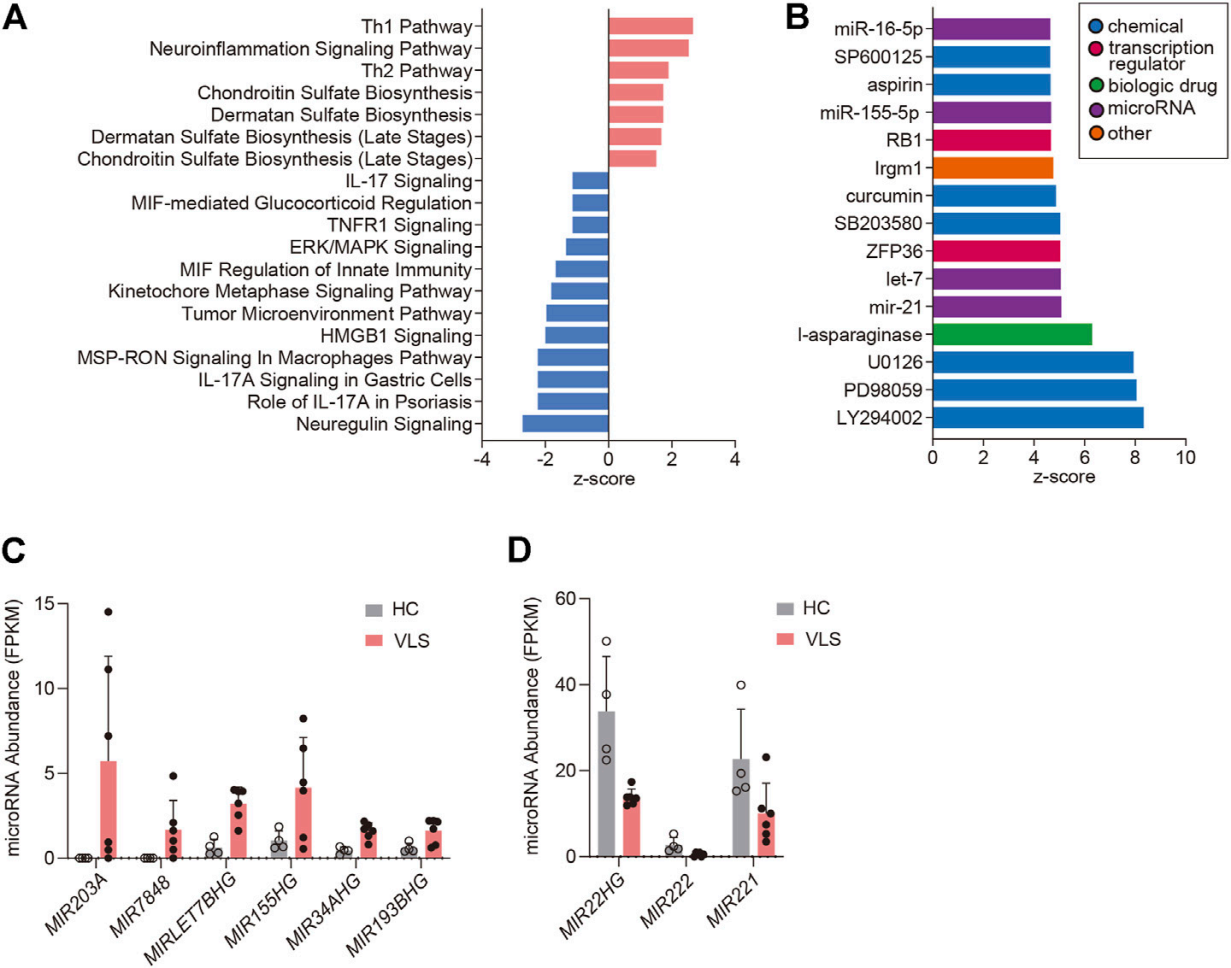
Pathway analysis and upstream analysis. **(A)** Enrichment of the top clusters using IPA GO algorithms for all significant DEGs. The threshold of minimum significance was set to FDR <0.05. The z-score predicts the direction of change for the function, and the threshold was set to an absolute value of z-score > 1. **(B)** Upstream Regulator Analysis. The top 15 activated upstream regulators ranked by z-scores are presented as a bar chart. **(C,D)** RNA-Seq data showing microRNA abundances in six VLS and four healthy samples. HC, Healthy Controls.

By using the Ingenuity Pathway Analysis (IPA) software, we performed Canonical Pathway Analysis and Upstream Regulator Analysis to unravel signaling pathways and key regulators implicated in VLS pathogenesis. Sixteen pathways were identified using an absolute z-score value greater than 1 as a threshold (FDR <0.05, Benjamini-Hochberg adjusted *p*-value) (Figure 3A). Among those, seven pathways were predicted to be activated (z > 1), and 12 pathways were predicted to be inhibited (z < -1). Both Th1 and Th2 pathways were found activated in VLS, reinforcing the role of T cell activation in VLS pathogenesis. However, pathways involved in interleukin-17 (IL-17) signaling, including “Role of IL-17A in Psoriasis”, “IL-17A Signaling in Gastric Cells”, and “IL-17 Signaling” were predicted to be inhibited (Figure 3A). RNA-seq data showed that expressions of *IL-17A* and downstream genes such as *CXCL1*, *CXCL3*, *CXCL8*, and *CCL20* (Blauvelt and Chiricozzi, 2018) were significantly downregulated in VLS. These data suggested that the balance of Th1/Th2 and Th17 Cells was skewed in VLS. Of note, pathways involved in the biosynthesis of glycosaminoglycans, including chondroitin sulfate and dermatan sulfate, were activated (Figure 3A). Glycosaminoglycans are major components of the extracellular matrix (ECM) (Theocharis et al., 2016), which could provide the tensile strength of the dermis. The alterations in glycosaminoglycans could be associated with ECM remodeling in VLS.

We then conducted Upstream Analysis with the DECGs by IPA. This analysis identifies potential upstream regulators driving the observed gene expression changes. IPA identified 230 activated potential upstream regulators (*p*-value < 0.05, |z-score| ≥ 3), and 15 top-ranked activated upstream regulators were demonstrated (Figure 3B). Chemicals that inhibit cell proliferation, including PI3K inhibitor LY294002 (z-score = 8.3) and MAPK pathway inhibitors such as PD98059 (z-score = 8.1, MEK inhibitor), U0126 (z-score = 7.9, MEK inhibitor), SB203580 (z-score = 5.0, p38 MAPK), SP600125 (z-score = 5.0, JNK inhibitor), all showed high positive z-scores, suggesting that cell growth inhibition is associated with VLS. Of note, MicroRNAs miR-21 (z-score = 5.1), let-7 (z-score = 5.1), miR-155-5p (z-score = 4.7), and miR-16-5p (z-score = 4.7) were predicted to be activated (Figure 3B). Promoted by these findings, we examined microRNA expressions in the DECG dataset. Genes encoding host microRNAs of miR-155 and let-7, i.e., *MIR155HG* and *MIRLET7BHG*, were upregulated (Figure 3C). This data correlates with the Upstream Analysis showing the activation of miR-155 and let-7 because intronic microRNAs are usually co-expressed with their host genes (Lutter et al., 2010). Moreover, the activation of miR-155 is consistent with a previous study reporting miR-155 upregulation in LS (Terlou et al., 2012). In addition to above mentioned two microRNAs, *MIR203A*, *MIR7848*, *MIR34AHG*, *MIR193BHG* were significantly upregulated in VLS, while *MIR22HG*, *MIR222*, and *MIR221* were downregulated (Figures 3C,D), suggesting that microRNAs homeostasis is involved in VLS.

### Network Analysis

**FIGURE 4:**
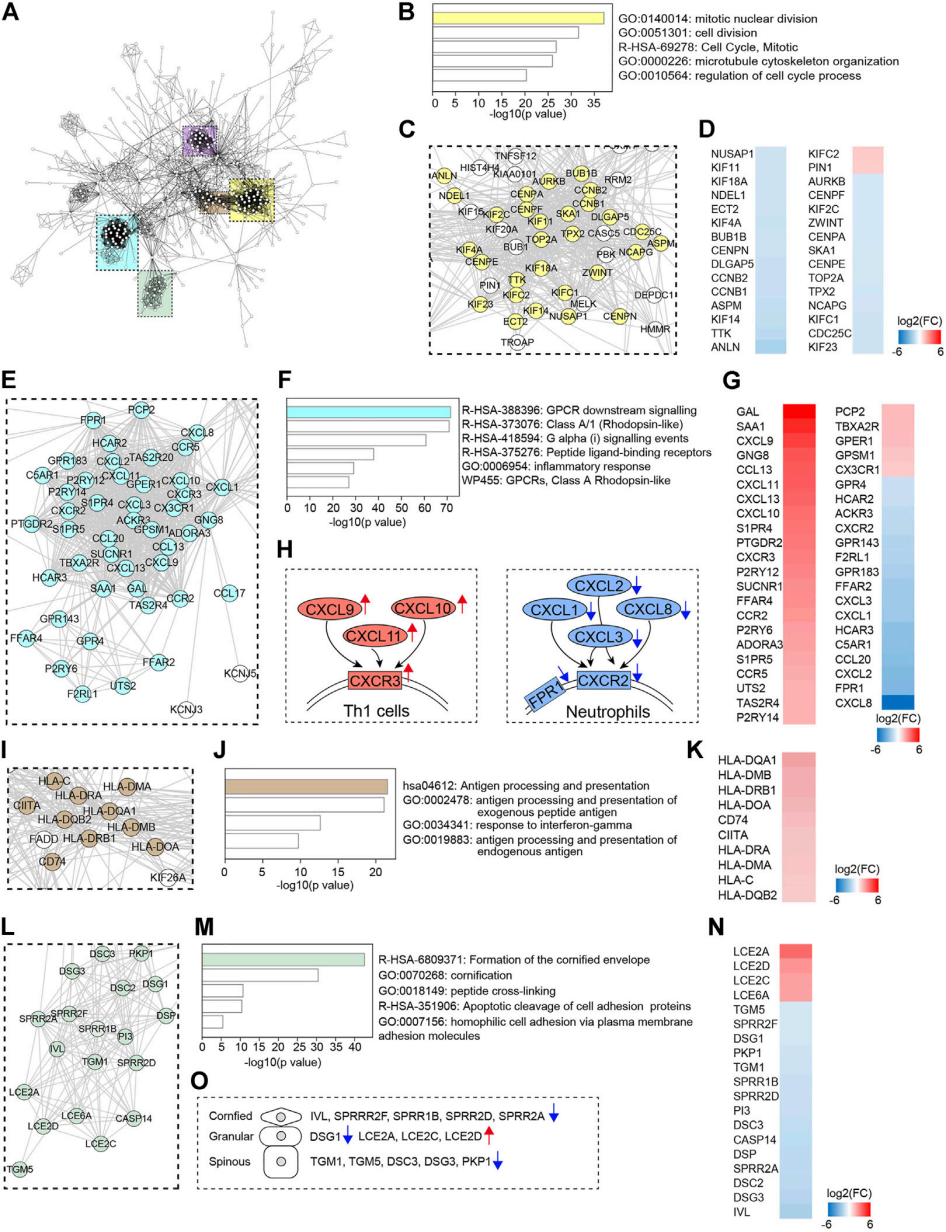
Protein-protein interaction (PPI) network analysis. **(A)** PPI network of DECGs identified in LS. Each node represents a protein, and interactions with confidence score >0.9 are presented. Colored rectangle boxes indicated subnetworks. **(B,F,J,M)** Metascape analysis of indicated subnetworks. **(C,E,I,L)** Subnetworks showing nodes labeled with gene name. Genes identified in the top category of Metascape analysis were labeled in colors. **(D,G,K,N)** Heatmaps showing fold changes of expression of genes labeled in colors. **(H)** Schematics showing gene expression changes of chemokine-chemokine receptor pairs. **(O)** Schematics showing changes in the expression of cornified envelope genes.

Next, by using STRING analysis (http://string-db.org/) (Szklarczyk et al., 2019), we built protein-protein interaction (PPI) networks with the DECGs in VLS. A dominant network composed of several densely connected subnetworks was generated using the confidence score of 0.9 (Figure 4A). We found that over 40 genes formed a subnetwork comprising genes predominantly involved in cell cycle regulation. Metascape analysis with these genes revealed that “mitotic nuclear division” is the top-ranked category (Figures 4B,C). Heatmap of gene expression level showed that the majority of the genes (28/30) were downregulated, including cyclin genes *CCNB1* and *CCNB2* and genes critical for centrosome formation and chromosome segregation (*CENPA*, *CENPE*, *CENPF*, and *CENPN*, and *AURKB*) (Petry, 2016) (Figure 4D). These data suggested that cell cycle progression was dysregulated in VLS. Another major subnetwork is composed of genes associated with “GPCR downstream signaling” (Figures 4E,F). In this subnetwork, genes were differentially regulated. CXCR (CXC chemokine receptor) is a group of GPCRs that reacts to the CXC chemokine family. We found that proinflammatory genes *CXCL9*, *CXCL10*, *CXCL11*, and their receptor *CXCR3*, were significantly upregulated (Figure 4G). The CXCL9, CXCL10, CXCL11/CXCR3 axis is important for T cell recruitment and activation (Tokunaga et al., 2018); thus, their upregulation could contribute to T cell infiltration in VLS (Figure 4H). In contrast, neutrophil chemoattractant genes *CXCL1*, *CXCL2*, *CXCL3*, *CXCL8*, and their cognate receptor *CXCR2* (Kolaczkowska and Kubes, 2013) were downregulated (Figure 4G), suggesting the inhibition of neutrophils in VLS (Figure 4H). We also found a subnetwork associated with antigen processing and presentation (Figures 4I,J), in which class II HLA genes *HLA-DQA1*, *HLA-DMB*, *HLA-DRB1*, *HLA-DOA*, *HLA-DRA*, *HLA-DMA*, and *HLA-DQB2*, as well as the class II–invariant chain *CD74* and transcriptional regulator *CIITA*, were upregulated (Figure 4K). This observation correlates with the reports that genes regulating HLA class II antigens are significantly associated with VLS pathogenesis (Marren et al., 1995). Of note, this analysis also formed another subnetwork related to antigen processing, which predominantly comprises genes participating in ubiquitination and proteasome degradation (Supplementary Figure S2). In addition, a subnetwork involved in cornified envelope formation was identified (Figures 4L,M). Genes encoding components of the cornified envelope (Candi et al., 2005), such as small proline-rich proteins (*SPRRR2F*, *SPRR1B*, *SPRR2D*, and *SPRR2A*), transglutaminases (*TGM1* and *TGM5*), desmogleins (*DSG1* and *DSG3*), and desmocollin (*DSC3*), were downregulated, while late cornified envelope family genes (*LCE2A*, *LCE2C*, *LCE2D*) were upregulated (Figures 4N,O). The cornified envelope locates at the outermost layer of the epidermis and functions as a mechanical barrier (Candi et al., 2005). Thus, the aberrant expression of cornified envelope genes may be responsible for the atrophy, fragility, and ecchymosis frequently observed in VLS.

### Validation of RNA-Seq Results

**FIGURE 5:**
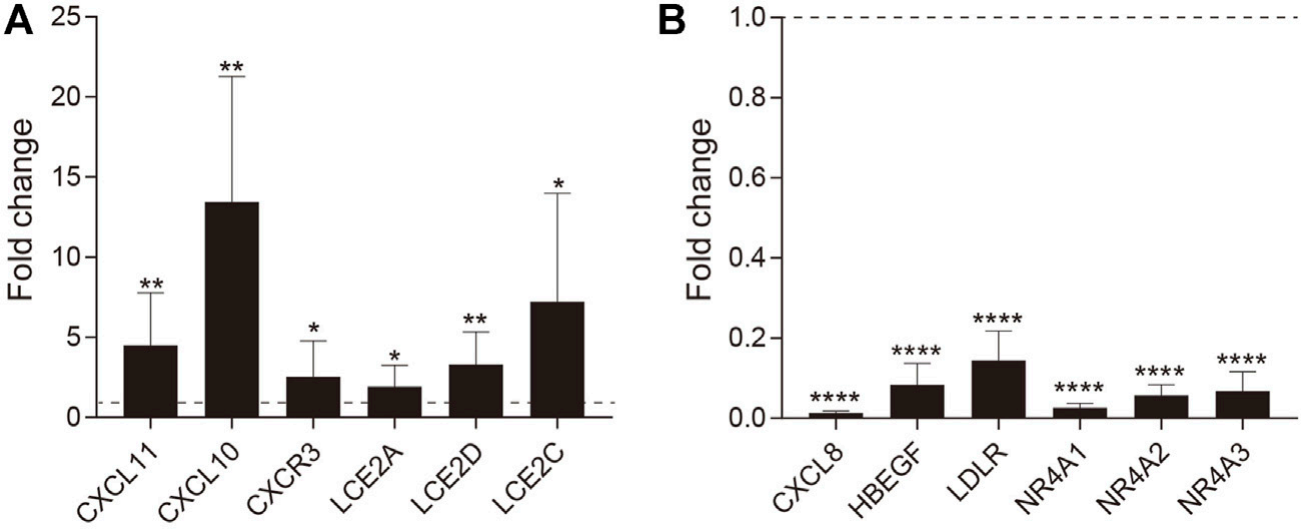
RT-qPCR validation of DEGs. The fold-change in expression is shown for **(A)** upregulated and **(B)** downregulated genes. Error bars represent the standard error of the mean (*p value ≤ 0.05, **p value ≤0.01, ****p value ≤0.0001).

We next verified the RNA-seq results by quantitative polymerase chain reactions (qPCR) analysis. Six upregulated and six downregulated genes identified by the RNA-seq analysis were tested. Of selected upregulated genes, *CXCR3*, *CXCL10*, and *CXCL11* were chemokine-chemokine receptor pairs involved in Th1 cell activation, and *LCE2A*, *LCE2C*, *LCE2D* are cornified envelope family genes specifically upregulated in VLS. Of down-regulated genes, *CXCL8* is the critical chemotactic factor for neutrophils, and *HBEGF*, *LDLR*, *NR4A1*, *NR4A2*, and *NR4A3* are randomly selected. Transcript levels for each gene were measured in comparison with *GAPDH* and then normalized to healthy control by using the 2^−ΔΔCt^ method. We found that all selected upregulated and downregulated genes in RNA-Seq were successfully validated by qPCR analysis (Figures 5A,B), indicating that the RNA-Seq analysis is reliable.

## Discussion

Complications of VLS mainly include pruritus, pain, sexual and/or urinary dysfunction, posing detrimental effects on quality of life. If not appropriately treated, VLS is associated with a greater degree of the lesion and an increased risk of genital cancer (Pugliese et al., 2007). To date, the etiology of VLS is elusive. Although efforts have been made to investigate disease mechanisms, there has been less research on VLS at the system level. Terlou et al. have investigated the pathogenesis of VLS and lichen planus by profiling gene expression using microarray analysis, and reveal microRNA-155, a microRNA involved in the regulation of the immune response, plays a role in immune dysregulation (Terlou et al., 2012); however, the regulatory network in VLS has not been fully explored. In the current study, we performed an RNA-seq-based transcriptome analysis of VLS lesions. This study revealed profound changes in expressions of both coding and non-coding genes. Moreover, critical signaling pathways and gene regulatory networks were depicted. These findings may shed light on the VLS pathogenesis and possible biomarkers for diagnosis and disease management.

VLS is considered lymphocyte-mediated dermatosis because one of the classic histological features is a lymphatic infiltrate belt underneath a condensed region of sclerosis (Tran et al., 2019). Corroborating this, we found that chemokine receptor genes *CXCR3* and *CCR5*, both of which are markers for Th-1 type reactions, as well as the TCR activation-associated molecules *CD3G*, *CD3D*, *ZAP70*, *LAT*, *LCK*, and *CD8B*, were significantly upregulated, suggesting a dominant CD8^+^ T cell response in VLS. Of note, the orphan nuclear receptor NR4A family proteins, including *NR4A1*, *NR4A2*, and *NR4A3,* are among top downregulated genes. This downregulation was further verified by our quantitative rt-PCR analysis. NR4A genes were recently suggested to be associated with T cell hyporesponsiveness, as evidenced by the fact that NR4A-depleted T cells exerted potent effector functions in the context of cancer and infection (Chen et al., 2019; Liu et al., 2019). As a result, we postulate that NR4A-deficient T cells in VLS lesions could have autoreactive activity, leading to autoimmune inflammation.

Intriguingly, we found that *CXCR2* gene, the major chemokine receptors on neutrophils, and its ligand genes *CXCL1*, *CXCL2*, *CXCL3*, and *CXCL8*, were significantly downregulated, suggesting the depletion of neutrophils in VLS. Neutrophils are the most abundant leukocytes in the circulation and play critical roles in immune and inflammatory responses (Kolaczkowska and Kubes, 2013). Infiltration of neutrophils has been observed in lichen planus (Atas et al., 2016), another T cell-mediated chronic skin disorder; however, to our knowledge, the roles of neutrophils in VLS have received little attention. Aside from its involvement in inflammation, neutrophils help wounds healing by directly regulating angiogenesis and cell proliferation (Phillipson and Kubes, 2019). Because skin bruising, tearing, and bleeding are typical in VLS (Fistarol and Itin, 2013), the absence of neutrophil-mediated wound heal could exaggerate these symptoms. In future studies, the presence of neutrophils in VLS lesions should be examined by histology approaches. Whether neutrophils could contribute to VLS pathogenesis or serve as a diagnostic biomarker needs to be investigated.

One of the noticeable features observed in the VLS transcriptome is the decreased expression of genes involved in cell cycle progression. These include cyclin genes *CCNB1*, *CCNB2*, *CCNL1*, *CCNE1*, *CCNK*. In contrast, only one cyclin gene, *CCNG2*, was upregulated in VLS. These data strongly suggested that cell cycle progression in LS lesions has been impeded. As a result, cell proliferation may be inhibited, resulting in skin atrophy and basal cell degeneration observed in VLS. Intriguingly, let-7, a microRNA known to repress cell proliferation (Johnson et al., 2007), was significantly upregulated in LS. Because let-7 could inhibit expressions of multiple cyclins, including *CCNB1*, *CCNE2*, *CCNF*, *CCNJ* in HepG2 cells (Johnson et al., 2007), it can be speculated that, in VLS, reduced expressions of cyclins are likely caused by let-7 upregulation. Of note, microRNA-203a, another significantly upregulated microRNA, also exerted potent anti-proliferative activity in multiple cells and systems (Chen et al., 2014; Deng et al., 2016; An and Zheng, 2020; Miao et al., 2020). Besides, genes encoding proteins in centromere formation and kinetochore assembly, including *CENPA*, *CENPE*, *CENPF*, *CENPN*, *BUB1B*, *AURKB*, and *SKA1*, were significantly downregulated. As centromere proteins play crucial roles in maintaining chromosome stability (Fukasawa, 2005; Petry, 2016), downregulation of these gene expressions could cause DNA damage and increased risk of malignancy in VLS.

In sum, our study provides a thorough gene expression profile and molecular interaction network for VLS, a disease with an elusive etiology. We revealed that coding genes, microRNAs, and long non-coding RNAs are profoundly dysregulated in VLS lesions. Furthermore, gene networks that regulate immune cell chemotaxis, mitotic nuclear division, antigen processing and presentation, or epidermal barrier construction were perturbed. These alterations may play crucial roles in the pathophysiology of VLS. It should be noted that the current study included a small number of cases, and a larger sample size need be used to generalize the findings. Future research with advanced methodologies, such as combining single-cell level transcriptomics with in-depth bioinformatics analysis, will shed further light on VLS pathogenesis and therapy.

## Data Availability

The datasets presented in this study can be found in online repositories. The names of the repository/repositories and accession number(s) can be found below: https://bigd.big.ac.cn/gsa, PRJCA007009.
