# Supplementary material for: Transcriptome Profiling and Network Analysis Provide Insights Into the Pathogenesis of Vulvar Lichen Sclerosus
Source: Front Genet. 2022 Jun 17;13:905450. doi: 10.3389/fgene.2022.905450 (PMC9247155; doi:10.3389/fgene.2022.905450)
Supplement: Supplementary file 1 [file Table1.DOCX]

**Table S1** Primer sequences for quantitative real-time reverse transcription-PCR

| **Gene** | **Forward primer** | **Reverse primer** |
| --- | --- | --- |
| CXCL8 | TGGCAGCCTTCCTGATTTCT | AATTTCTGTGTTGGCGCAGT |
| HBEGF | TTTCTGGCTGCAGTTCTCTC | AGGTCCAGATCTGCCTCTTG |
| LDLR | TCGCCTACCTCTTCTTCACC | CTGGGTGCTGCAGATCATTC |
| NR4A1 | GCCAATCTCCTCACTTCCCT | CAGCTCCTGGAACTTGGAGT |
| NR4A3 | TCCATCAGGTCAAACACTGC | CAAATCCACGAAGGCACTGA |
| NR4A2 | AGAGAAGATCCCTGGCTTCG | CCACTGGGTTGGACCTGTAT |
| CXCL11 | AAGGTGGGTGAAAGGACCAA | TCCGATGGTAACCAGCCTTT |
| CXCL10 | AGTGGCATTCAAGGAGTACCT | TGATGGCCTTCGATTCTGGA |
| CXCR3 | AGGTGCCCTCTTCAACATCA | GCTGGGTGGCATGAACTATG |
| LCE2A | ACCTCGAACATCACAGAGCA | CTCTTGGGCCATGACAACAG |
| LCE2D | AAACCTGCTACAGCCTGATG | GGTGCTCCATCAAGCACAAA |
| LCE2C | TGGGACTGAATGGCCAAGAA | CTTTGTGGTCTCTGCCAACC |
