# Supplementary figures and images for: Transcriptome Profiling and Network Analysis Provide Insights Into the Pathogenesis of Vulvar Lichen Sclerosus
Source: Front Genet. 2022 Jun 17;13:905450. doi: 10.3389/fgene.2022.905450 (PMC9247155; doi:10.3389/fgene.2022.905450)

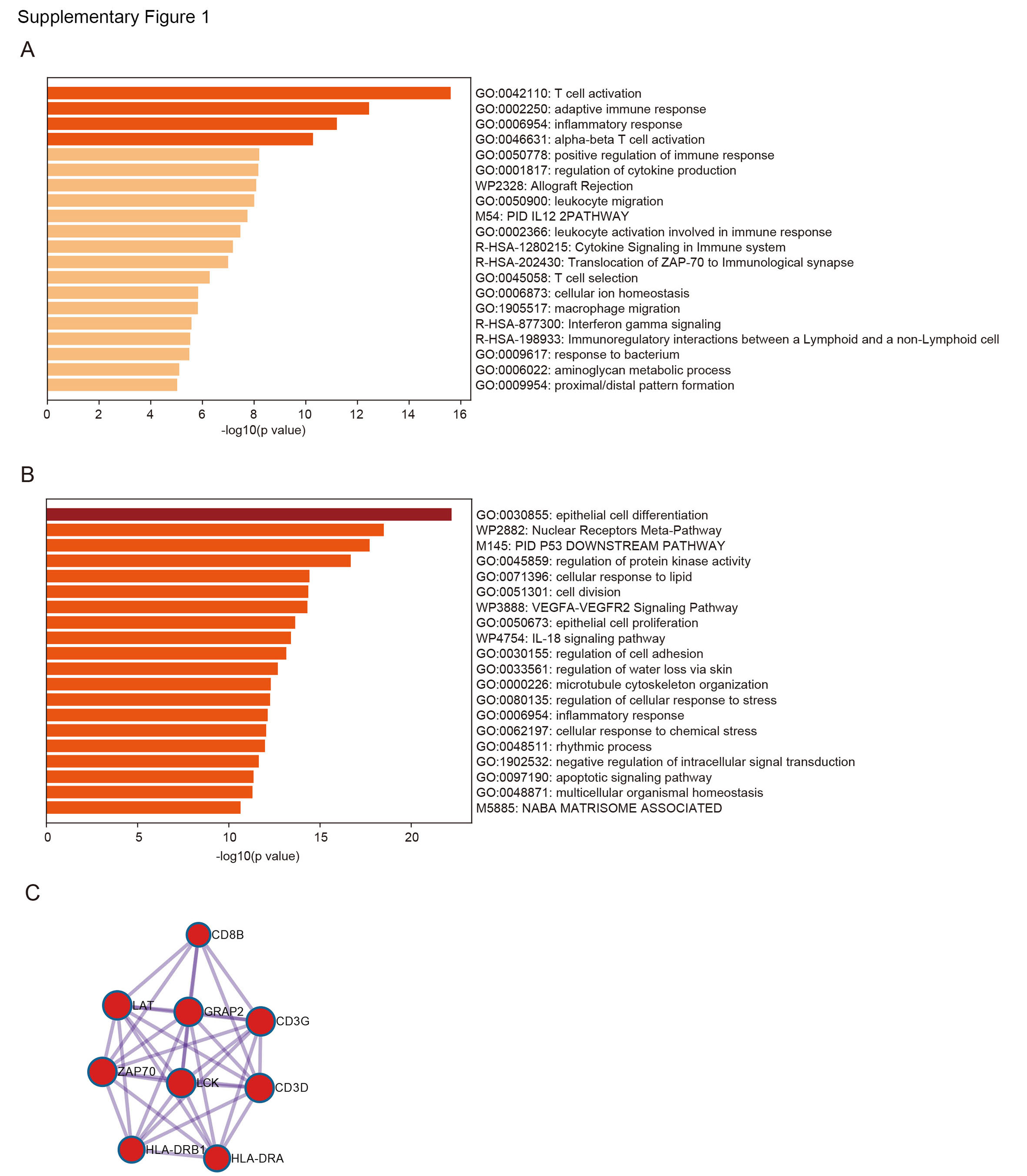

Supplement: Supplementary file 2 [file Image1.JPEG]

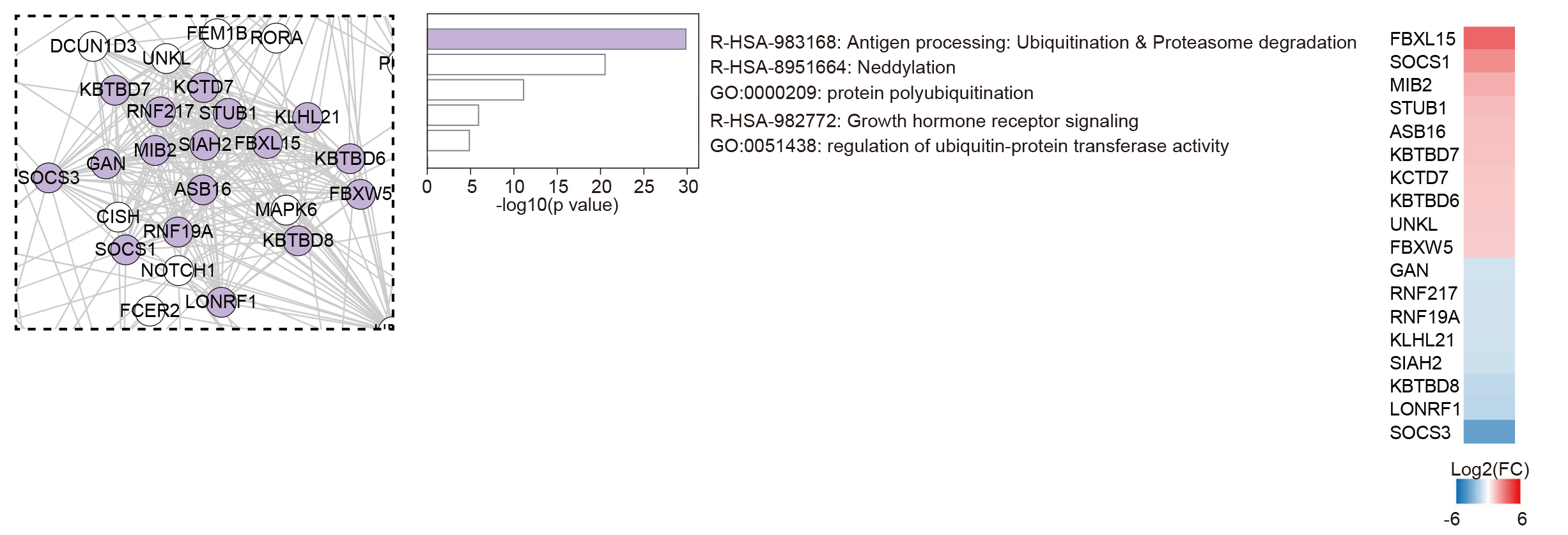

Supplement: Supplementary file 3 [file Image2.JPEG]
