# Supplementary material for: Transcriptome Profiling and Network Analysis Provide Insights Into the Pathogenesis of Vulvar Lichen Sclerosus
Source: Front Genet. 2022 Jun 17;13:905450. doi: 10.3389/fgene.2022.905450 (PMC9247155; doi:10.3389/fgene.2022.905450)
Supplement: Supplementary file 4 [file Table2.docx]

|  | VLS patient (n = 6)  Mean ± SD | Healthy control (n = 4)  Mean ± SD |
| --- | --- | --- |
| Age | 55.5 ± 20 | 52.8 ± 23.2 |
| Sex (Male: Female) | 0:6 | 0:4 |
| Age of first onset | 39.5 ± 17.2 | - |

Supplementary Table 2. The demographic characteristics of the VLS patients
