# Supplementary material for: Transcriptome Profiling and Network Analysis Provide Insights Into the Pathogenesis of Vulvar Lichen Sclerosus
Source: Front Genet. 2022 Jun 17;13:905450. doi: 10.3389/fgene.2022.905450 (PMC9247155; doi:10.3389/fgene.2022.905450)
Supplement: Supplementary file 6 [file Table3.DOCX]

Supplementary Table 3. The clinical characteristics of the VLS patients

| Patient | Smoking | Diagnostic delay (Years) | Fusion (Agglutination) | Lichenification | Loss of vulval architecture | Ulceration | Purpura | Itching | BMI |
| --- | --- | --- | --- | --- | --- | --- | --- | --- | --- |
| P1 | None | 7 | Moderate | Moderate | Moderate | None | Mild | Moderate | 23.4 |
| P2 | None | 10 | Moderate | Moderate | Moderate | None | None | severe | 22.4 |
| P3 | None | 10 | Very severe | Severe | Very severe | None | Mild | Moderate | 25.4 |
| P4 | None | 8 | Moderate | Moderate | severe | None | Mild | Very severe | 18.6 |
| P5 | None | 30 | Mild | Mild | Mild | None | None | Moderate | 22 |
| P6 | None | 1 | Severe | Moderate | Severe | None | None | Moderate | 21.5 |
